# The McGurk effect is similar in native Mandarin Chinese and American English speakers

**DOI:** 10.3389/fpsyg.2025.1531566

**Published:** 2025-03-28

**Authors:** John F. Magnotti, Debshila Basu Mallick, Guo Feng, Bin Zhou, Wen Zhou, Michael S. Beauchamp

**Affiliations:** ^1^Department of Neurosurgery, University of Pennsylvania Perelman School of Medicine, Philadelphia, PA, United States; ^2^OpenStax, Rice University, Houston, TX, United States; ^3^Psychological Research and Counseling Center, Southwest Jiaotong University, Chengdu, Sichuan, China; ^4^Key Laboratory of Mental Health, Institute of Psychology, Chinese Academy of Sciences, Beijing, China

**Keywords:** McGurk effect, cultural differences, audiovisual speech, multisensory integration, individual differences

## Abstract

Humans combine the visual information from mouth movements with auditory information from the voice to recognize speech. A common method for assessing audiovisual speech perception is the McGurk effect: when presented with some incongruent pairings of auditory and visual speech syllables (e.g., the auditory speech sound “ba” dubbed onto the visual mouth movements for “ga”) individuals perceive a third syllable, distinct from the auditory and visual components. The many differences between Chinese and American culture and language suggest the possibility of group differences in the McGurk effect. Published studies have reported less McGurk effect in native Mandarin Chinese speakers than in English speakers, but these studies sampled small numbers of participants tested with a small number of stimuli. Therefore, we conducted in-person tests of the McGurk effect in large samples of Mandarin-speaking individuals from China and English-speaking individuals from the USA (total *N* = 307) viewing nine different stimuli. Averaged across participants and stimuli, we found similar frequencies of the McGurk effect between Chinese and American participants (48% vs. 44%). In both groups, there was high variability both across participants (range from 0% to 100%) and stimuli (14%−83%) with the main effect of culture and language accounting for only 0.2% of the variance in the data. The high variability inherent to the McGurk effect necessitates the use of large sample sizes to accurately estimate group differences and requires testing with a variety of McGurk stimuli, especially stimuli potent enough to evoke the illusion in the majority of participants.

## Introduction

Humans around the world communicate by speaking and listening face-to-face. During these interactions, we integrate the heard speech sounds with the seen mouth movements to increase both the speed and accuracy of speech perception (Peelle and Sommers, 2015; Ross et al., 2007; Sumby and Pollack, 1954; van Wassenhove et al., 2005). A common way to assess multisensory integration during speech perception is an illusion known as the McGurk effect (McGurk and MacDonald, 1976) in which individuals presented with incongruent auditory and visual syllables report hearing an entirely different syllable. The McGurk effect has become a popular assay of multisensory speech perception because it is easy to administer: both the stimulus and the response consist of only a single syllable. However, some individuals do not experience the effect and instead perceive the auditory or visual components of the stimulus (Nath and Beauchamp, 2012; Stevenson et al., 2012). These individual differences are consistent across test-retest intervals of 12 months or longer, suggesting that they reflect stable differences in the propensity to integrate auditory and visual speech information (Basu Mallick et al., 2015).

Although many laboratory studies of psychological phenomena focus exclusively on native English speakers, the McGurk effect is an important exception. It has been studied across native speakers of Mandarin Chinese, Cantonese, Thai, and Japanese (Burnham and Lau, 1998; Chen and Hazan, 2007; Sekiyama, 1997; Sekiyama and Tohkura, 1991), Spanish, German, Hungarian (Fuster-Duran, 1996; Grassegger, 1995), Italian (Bovo et al., 2009), Finnish (Sams et al., 1998; Traunmüller and Öhrström, 2007), and Hebrew (Aloufy et al., 1996). The groups in these studies are defined both by cultural differences and by differences in their native language; in this paper, we group them and refer to them together as “intercultural.”

The strongest claim in the literature for intercultural differences in the McGurk effect involves comparisons between Asian and non-Asian cultures. Sekiyama and Tohkura (1991, 1993) reported a lower frequency of McGurk perception in native Japanese speakers than in native English speakers, and equal or lower frequency in Mandarin Chinese speakers than in Japanese speakers (Hayashi and Sekiyama, 1998; Sekiyama, 1997). In agreement with these results, Burnham and Lau (1998) found a lower frequency of McGurk perception in Cantonese speakers than English speakers although other studies comparing English and Chinese speakers did not find differences in McGurk frequency (Chen and Hazan, 2007, 2009).

Two major groups of hypotheses have emerged to explain intercultural differences in the McGurk effect. The linguistic hypothesis explains them via the properties of Asian languages. Tonal languages (such as Mandarin) and semi-tonal languages (such as pitch accents in Japanese) may increase reliance on auditory speech cues, decreasing the relevance of visual speech information (Sekiyama, 1997). Phonemes of Mandarin and Japanese may be easier to discriminate without visual cues than those of English, reducing the need for visual speech information to disambiguate speech sounds (Sekiyama and Burnham, 2008). The face-avoidance hypothesis explains them via the cultural milieu of the listener. In Japanese and Chinese cultures, direct viewing of the face can be considered impolite and hence may discourage people in these cultures from developing a strong reliance on the visual speech information required for perception of the McGurk effect (Sekiyama, 1997). There is some evidence that English-speaking children are better at visual-only identification of speech than Japanese children (Sekiyama and Burnham, 2008).

One potential problem with these findings of intercultural differences is that they were conducted before recent advances in our understanding of individual differences in the McGurk effect. Some native English speakers never perceive the illusion and others always perceive it (Magnotti and Beauchamp, 2015; Basu Mallick et al., 2015; Nath and Beauchamp, 2012; Stevenson et al., 2012; Strand et al., 2014). High variability means that large sample sizes are necessary for accurate statistical inference, but many studies of cultural differences in the McGurk effect have used small sample sizes (e.g., 10–14 participants, Bovo et al., 2009; Sekiyama, 1994, 1997), possibly resulting in inferential errors (Magnotti and Beauchamp, 2018).

Another difficulty in interpreting the literature is that stimuli from different talkers (or even different stimuli from the same talker) vary greatly in their ability to evoke the McGurk effect (Jiang and Bernstein, 2011; Magnotti and Beauchamp, 2015; Basu Mallick et al., 2015). This variability is problematic when cross-cultural studies use stimuli created from only two talkers (Bovo et al., 2009; Burnham and Lau, 1998; Hayashi and Sekiyama, 1998; Sekiyama, 1994, 1997; Sekiyama and Tohkura, 1993). Just as testing a small group of participants from a highly variable population is problematic, testing only a few McGurk stimuli can also lead to errors in inference due to the idiosyncrasies of individual stimuli, especially if only weak stimuli that rarely evoke the McGurk effect are tested.

To overcome these difficulties, we compared McGurk perception between a large sample of Mandarin-speaking individuals from China (*n* = 162) and a large sample of English-speaking individuals from the USA (*n* = 145) using a battery of nine McGurk stimuli from eight different talkers. In-person testing with a large sample of participants and stimuli allowed for the accurate estimation of intercultural differences in the McGurk effect.

## Methods

### Chinese participants

All participants gave written informed consent to participate in an experimental protocol approved by the Institutional Review Board of the Institute of Psychology of the Chinese Academy of Sciences. Parental informed consent was obtained for participants under 18 years of age. Participants were tested in-person and consisted of *n* = 162 Mandarin speakers native to China (82 female; mean age = 17 years, range = 14–23) recruited from the Beijing Twin Study project of the Institute of Psychology of Chinese Academy of Sciences (analysis was only conducted on the first-born of each twin pair). All participants reported normal or corrected-to-normal vision and no history of speech, language, or hearing difficulties.

### American participants

All participants gave written informed consent to participate in an experimental protocol approved by the Institutional Review Board of Rice University. All participants were native to the USA and reported English as their primary language (*n* = 145; 97 female, mean age = 19 years, range = 18–26). All participants reported normal or corrected-to-normal vision and no history of speech, language, or hearing difficulties, and were tested in-person.

### Data and code availability

Data and analysis code are available in the Supplementary material.

### Stimuli and procedure

The McGurk stimuli consisted of nine audiovisual recordings, lasting 2 s each. Each stimulus contained an auditory recording of a syllable and a video recording of the face of the same talker enunciating a different syllable. Four stimuli consisted of auditory “ba” and visual “ga” (AbaVga). Three stimuli consisted of double syllables, auditory “baba” paired with visual “gaga” (AbabaVgaga). Two stimuli consisted of auditory “pa” and visual “ka” (ApaVka). There were five male speakers and three female speakers (the same female speaker appeared in two stimuli). Stimuli were viewed at a distance of 40 cm and filled a 15″ LCD display.

During the experiment, the stimuli were presented in random order. Participants in the China group saw each McGurk stimulus eight times; participants in the USA group saw each McGurk stimulus 10 times, but we analyzed only the first eight presentations to match the China group (the results were unchanged when all 10 presentations were analyzed).

Participants reported their percepts by speaking aloud and no feedback was given. Responses were recorded by the stimulus computer and transcribed by a research assistant. The USA group also viewed control stimuli (10 times each) intermixed with the McGurk stimuli: six congruent audiovisual syllables (“ba,” “ga,” “pa,” “ka,” “da,” “ta”) and two non-McGurk incongruent stimuli, which are similar to McGurk stimuli, but with the auditory and visual constituents reversed (AgaVba and AkaVpa) all spoken by the same female speaker.

### Scoring responses

Responses to McGurk stimuli were categorized as follows. The responses “da” or “tha” (to AbaVga) and “ta” or “tha” (to ApaVka) were categorized as McGurk fusion responses. The responses “ba” (to AbaVga) and “pa” (to ApaVka) were categorized as auditory responses. The responses “ga” (to AbaVga) and “ka” (to ApaVka) were categorized as visual responses. Any other response was categorized as “other.” For AbabaVgaga stimuli, each syllable was coded separately (e.g., the response “dada” was coded as 1.0 McGurk; the response “bada” was coded as 0.5 McGurk and 0.5 auditory).

Across all subjects and stimuli, the McGurk responses (46%) and auditory responses (37%) were the most common. Visual responses (7%) and “other” responses (10%) were comparatively rare across stimuli and individuals. Only two stimuli had visual responses more than 15% of time, and only two stimuli had “other” responses more than 15% of time. This pattern of responding led to complementary percentages between McGurk and auditory responses, and thus we analyzed only McGurk responses to each stimulus.

## Results

**Figure 1:**
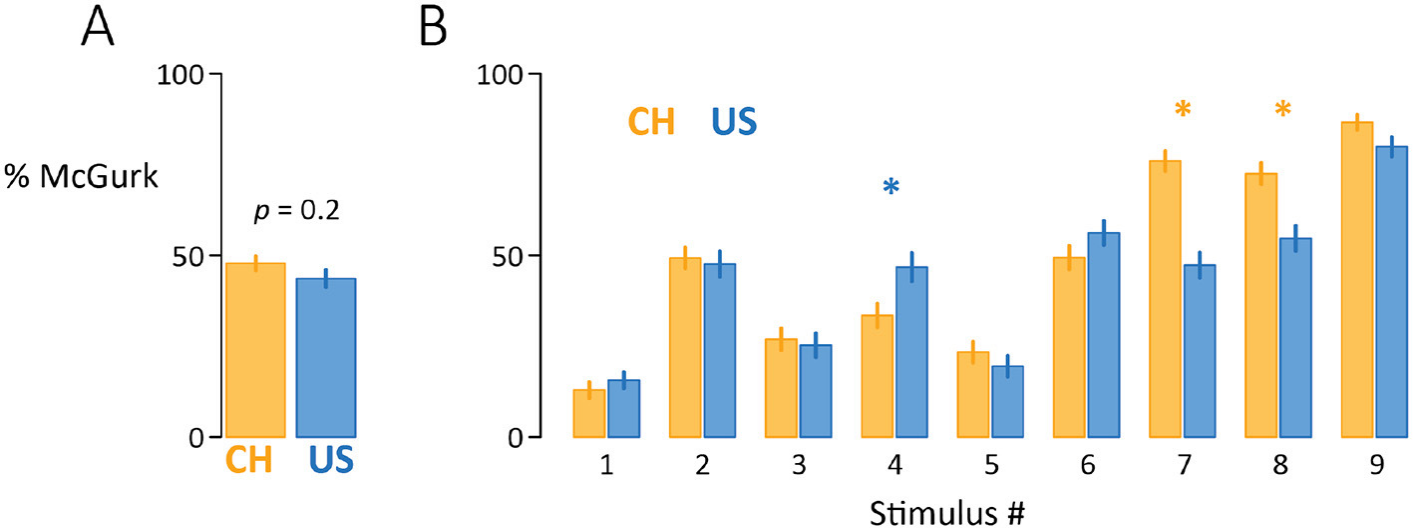
**(A)** Overall percent of McGurk fusion responses for native Mandarin speakers from China (*n* = 162; orange) and native English speakers from the USA (*n* = 145; blue). Reported *p*-value is from a linear mixed-effects model comparing McGurk percentage between groups. **(B)** McGurk percentage for each stimulus and group. Stimuli are arranged by overall McGurk percentage. Asterisks indicate significant differences between groups (*p* < 0.05). Stimulus #1 *(S1)*: Female talker (F); auditory *ba* with visual *ga* (A*ba*V*ga*); *S2*: F, A*baba*V*gaga*; *S3*: M, A*baba*V*gaga*; *S4*: M, A*baba*V*gaga*; *S5*: M, A*ba*V*ga*; *S6*: M, A*ba*V*ga*; *S7*: M, A*ba*V*ga*; *S8*: F, A*pa*V*ka*. *S9*: F, A*pa*V*ka*. *S1* and *S8* were the same talker.

We compared the frequency of the McGurk effect in native Mandarin-speaking individuals from China and native English-speaking individuals from the USA across nine stimuli (Figure 1A). The overall frequency of McGurk responses for the China group (Mean = 48%, standard error of the mean, SEM = 2%) was slightly greater than the USA group (Mean = 44%, SEM = 2%). In both groups, there was high variability across participants, with some participants in both groups never perceiving the illusion (0%) and some from both groups always perceiving the illusion (100%). Stimuli varied dramatically in their effectiveness (Figure 1B). The weakest stimulus (#1 in the ranking) evoked the McGurk effect on only 14% of trials (averaged across all participants), while the strongest stimulus (#9) evoked the McGurk effect on 83% of trials (Figure 1B).

A linear mixed-effects model was fit with percent McGurk responses as the dependent measure, fixed factors of cultural group and stimulus, and random effect of participant. There was no main effect of cultural group [$X_{\left(1\right)}^{2}$ = 1.9, *p* = 0.17]. There was a main effect of stimulus [$X_{\left(8\right)}^{2}$ = 1597.4, *p* < 10^−16^], driven by the wide range of effectiveness across stimuli, and a significant stimulus-by-cultural group interaction [$X_{\left(8\right)}^{2}$ = 124.4, *p* < 10^−16^] driven by greater McGurk for the USA group for stimulus 4 [*post-hoc t* = −3.1, *p* = 0.002], and greater McGurk for the China group for stimulus 7 [*t* = 6.7, *p* = 10^−11^] and stimulus 8 [*t* = 4.2, *p* = 10^−5^]; other stimuli showed no difference (all *p*s > 0.1).

There were no obvious explanations for the differences between stimuli. Stimulus #4 (USA > CH) was a male talker saying A*baba*V*gaga*, while stimulus #7 (USA < CH) was a male talker saying A*ba*V*ga* and stimulus #8 (USA < CH) was a female talker saying A*pa*V*ka*. Two stimuli recorded by the same female talker showed different response profiles, stimulus #1 (USA ~= CH) and stimulus #8 (USA < CH).

## Discussion

In a sample of 307 individuals and nine stimuli, similar frequencies of the McGurk effect were observed in native Mandarin speakers from China and native English speakers from the USA. In each group, there was high variability across participants (range from 0% to 100%) and stimuli (14%−83%). The large sample size allowed for the accurate estimation of effect sizes. The main effect of cultural group accounted for only 0.2% of variance in the frequency of McGurk perception; the interaction between cultural group and stimulus accounted for only 2% of the variance.

The finding of similar frequencies of McGurk perception in native Mandarin and native English speakers supports other evidence that the fundamentals of speech perception are similar between the two groups (Chen and Hazan, 2009; Hazan et al., 2010) but fail to replicate previous reports of significantly reduced susceptibility to the McGurk effect in native Chinese speakers (Burnham and Lau, 1998; Hayashi and Sekiyama, 1998; Sekiyama, 1997).

Studies that reported significant intercultural differences in the McGurk effect in native Chinese speakers tested many fewer participants than the present study, which did not find a significant intercultural effect. The pattern of a small study or studies showing significant effects followed by a null result from a well-powered study is not uncommon in the McGurk literature. In native Japanese speakers, studies with small sample sizes reported little or no McGurk effect (Sekiyama and Tohkura, 1991, 1993) but two larger studies refuted this claim (Magnotti et al., 2024a; Tiippana et al., 2023). A small study claimed that skilled musician did not experience the McGurk effect (Proverbio et al., 2016), while larger studies reached a very different conclusion (Lee et al., 2024; Politzer-Ahles and Pan, 2019). Small studies reported differences in the McGurk effect between autistic and non-autistic adults but a highly-powered study (*n* = 869) found no difference (Jertberg et al., 2024).

A likely explanation for this pattern is that the large variability in individual susceptibility to the McGurk effect makes it impossible to precisely estimate McGurk frequency using the small sample sizes (<15 per group) typical of many McGurk group difference studies. A study with 15 participants per group would have only 18% power to detect a 10% difference in the frequency of the McGurk effect between groups (Magnotti and Beauchamp, 2018). In contrast, the current study (with an ~10 times larger *n*) had 83% power to find group differences as small as 10%. Finding a significant result with a small sample size is sometime taken as evidence that the effect must be robust, but this is a fallacy (Loken and Gelman, 2017). Instead, it usually reflects a “winner's curse” where low power, publication bias, and a multiverse of data analysis choices combine to produce inflated estimates of effect size (Ferguson and Heene, 2012; Kuhberger et al., 2014; Lindstromberg, 2023; Magnotti and Beauchamp, 2018; Steegen et al., 2016).

Along with large sample sizes, an equally important ingredient for reproducibility is quantitative models that make specific predictions. As an example, in the Bayesian framework for multisensory integration, the contribution of a sensory modality to perception is inversely proportional to its reliability (Angelaki et al., 2009; Ernst and Banks, 2002). Applied to the McGurk effect, the Bayesian framework predicts that decreasing the reliability of auditory speech should increase the perceptual weighting of visual speech, resulting in increased McGurk effect (Magnotti and Beauchamp, 2017, 2015). Consistent with this hypothesis, in a clinical population with profound hearing loss (cochlear implant users), greater susceptibility to the McGurk effect was observed than in controls (Stropahl et al., 2017). Age-related hearing loss in ubiquitous in older adults, and, as expected, the McGurk effect increased with age in a study with participant ages ranging from 18 to 75 years (Jertberg et al., 2024). The reliability of the auditory modality can be decreased experimentally by adding auditory noise to the stimulus, and, as predicted, this increased the McGurk effect within individual participants (Fixmer and Hawkins, 1998; Stacey et al., 2020).

In summary, individual variability in the McGurk effect necessitates large sample sizes (>50 participants per group) to accurately estimate group differences (Magnotti and Beauchamp, 2018). Variability across McGurk stimuli necessitates testing with a variety of McGurk stimuli, especially stimuli that are highly effective in evoking the effect (Magnotti et al., 2024b).

## Author's note

This study was originally published with the title “Similar frequency of the McGurk effect in large samples of native Mandarin Chinese and American English speakers” (Magnotti et al., 2015). The original publication showed images (still frames) from the McGurk video stimuli. To protect the privacy of those shown in the images, in 2024 the original publication was retracted and removed from the publisher's website, with the agreement of the authors. Because the retraction was unrelated to the study data, the study was republished in the present manuscript without the McGurk stimulus images to allow the findings to remain available to the scientific community. The present manuscript was given a new title to avoid confusion with the retracted publication.

## Data Availability

The raw data supporting the conclusions of this article will be made available by the authors, without undue reservation.
