# Supplementary material for: The McGurk effect is similar in native Mandarin Chinese and American English speakers
Source: Front Psychol. 2025 Mar 28;16:1531566. doi: 10.3389/fpsyg.2025.1531566 (PMC11987121; doi:10.3389/fpsyg.2025.1531566)
Supplement: Supplementary file 1 [file Supplementary_file_1.zip › full_results_02_18_25.html]

The McGurk effect is similar in native Mandarin Chinese and American English speakers


Code 

- Show All Code
- Hide All Code

# The McGurk effect is similar in native Mandarin Chinese and American English speakers

#### Magnotti, Basu Mallick, Feng, Zhou, Zhou, & Beauchamp

- 1 Code setup
  - 1.1 Helper functions
- 2 Load data
- 3 Compare Groups
  - 3.1 Overall comparison
  - 3.2 Stimulus level
    comparision
  - 3.3 LME on group x stimulus

# 1 Code setup

```
stopifnot(all(
  suppressMessages(sapply(c('lme4', 'car', 'readxl', 'magrittr', 'knitr', 'emmeans','MuMIn'), 
         require, quietly = TRUE, character.only=TRUE))
))

knitr::opts_chunk$set(eval = TRUE, fig.align = 'center', class.source='fold-hide')
```

## 1.1 Helper functions

```
# kable doesn't like matrix output from aggregate
fix_agg_columns <- function(m) {
  to_fix <- which(sapply(m, is.matrix))
  for(ii in seq_along(to_fix)) {
    mat = m[[to_fix[ii]]]
    for(v in colnames(mat)) {
      m[[paste0(names(to_fix)[ii], '_', v)]] = mat[,v]
    }
  }
  m[to_fix] = NULL
  return(m)
}


rbind_list <- function (ll) do.call(rbind, ll)

do_aggregate <- function(d, ...) {
  aggregate(..., data=d)
}

ruta_axis <- function (side, at, tcl = -0.3, labels = at, las = 1, cex.axis = 1.2,
                       cex.lab = 1.2, mgpy = c(3, 0.6, 0),
                       mgpx = c(3, 0.75, 0), ...)  {
  if (length(side) > 1) {
    return(invisible(sapply(side, ruta_axis, at = at, tcl = tcl,
                            labels = labels, cex.axis = cex.axis, las = las,
                            cex.lab = cex.lab, ...)))
  }
  mgp <- mgpy
  if (side%%2)
    mgp <- mgpx
  invisible(as.matrix(axis(side, at = at, labels = labels,
                           tcl = tcl, mgp = mgp, cex.axis = cex.axis, las = las,
                           cex.lab = cex.lab, ...)))
}


as_pdf <- function (fname, w, h, expr, to_file = FALSE, bg = "white")  {
  if (to_file) {
    on.exit(dev.off())
    fname <- fix_pdf_name(fname)
    pdf(fname, width = w, height = h, useDingbats = FALSE, 
        bg = bg)
    res = eval(expr)
  }
  else {
    res = eval(expr)
  }
  return(invisible(res))
}

fix_pdf_name <- function (fname)  {
  if (!grepl("\\.pdf$", fname)) {
    fname = paste0(fname, ".pdf")
  }
  return(fname)
}

lighten <- function(col, amt=0.5) {
  if(length(col) > 1) return(sapply(col, lighten, amt=amt))
  
  # cc = c(col2rgb(adjustcolor(col, amt), alpha = TRUE))
  
  cc <- c(col2rgb(col))
  
  new_color <- 255 * (
    cc/255 * (amt) + c(1,1,1)*(1-amt)
  )
  
  rgb(new_color[1], new_color[2], new_color[3], maxColorValue = 255)
}


ebars.y <- function (x, y, sem, length = 0.05, up = T, down = T, code = 0, lwd=2,
                     ...) {
  if (up) {
    arrows(x0 = x, y0 = as.numeric(y), y1 = as.numeric(y + 
                                                         sem), angle = 90, code = code, length = length, lwd=lwd, ...)
  }
  if (down) {
    arrows(x0 = x, y0 = as.numeric(y), y1 = as.numeric(y - 
                                                         sem), angle = 90, code = code, length = length, lwd=lwd, ...)
  }
}
ebars <- ebars.y

m_se <- function (x)  {
  if (length(x) == 1) 
    return(c(mean = x, se = 0))
  c(mean = mean(x), se = se(x))
}

not_NA <- function(x) !is.na(x)

se <- function(x, na.rm=TRUE) {
  n <- sum(not_NA(x))
  
  re <- if (n < 2) {
    0
  } else {
    stats::sd(x, na.rm = na.rm)/sqrt(n)
  }
  
  if (is.na(re)) {
    re <- 0
  }
  
  return(re)
}

colors =c('us'='dodgerblue3', 'ch'='orange')
```

# 2 Load data

```
fusion_data <- read.csv('us_ch_compare_mcg.csv')

# make pct fusion variable
fusion_data$pct_fusion = 100*fusion_data$fusion

head(fusion_data)
```

```
##   movie fusion subject_id language syllables syllable_count pct_fusion
## 1     1      0          1       CH        BG              1          0
## 2     1      0          2       CH        BG              1          0
## 3     1      0          3       CH        BG              1          0
## 4     1      0          4       CH        BG              1          0
## 5     1      0          5       CH        BG              1          0
## 6     1      0          6       CH        BG              1          0
```

# 3 Compare Groups

## 3.1 Overall comparison

```
# collapse across stimulus w/n subject, then across subject to get mean and SE
subject_agg <- aggregate(
  pct_fusion ~ subject_id + language,
  function(x) round(d=1, mean(x)),
  data=fusion_data
)

agg <- aggregate(pct_fusion ~ language, m_se, data=subject_agg)

kable(fix_agg_columns(agg))
```

| language | pct\_fusion\_mean | pct\_fusion\_se |
| --- | --- | --- |
| CH | 47.83457 | 1.903218 |
| US | 43.65793 | 2.397675 |

```
cc <- colors[c('ch', 'us')]


as_pdf('./barplot_overall_fusion', w=1.33, h=2.5, {
  par(mar=c(2,3,1,1))
  xp <- barplot(
    agg$pct_fusion[,1], border=cc, col=lighten(cc, 2/3),
    ylim = c(0,100), axes=F
  )
  ruta_axis(2, 0:2*50)
  legend('topleft', legend=c('CH', 'US'), text.col = colors[c('ch', 'us')], cex=2, bty='n')
  ebars.y(xp, agg$pct_fusion[,1], agg$pct_fusion[,2], col=cc)
})
```

```
# get the range across participants
kable(caption='Range across participants', 
      fix_agg_columns(aggregate(pct_fusion ~ language, function(x)c('min'=min(x), 'max'=max(x)),
    data=subject_agg
)))
```

Range across participants

| language | pct\_fusion\_min | pct\_fusion\_max |
| --- | --- | --- |
| CH | 0 | 100 |
| US | 0 | 100 |

## 3.2 Stimulus level comparision

```
stim_agg <- aggregate(
  pct_fusion ~ movie,
  function(x) round(d=1, m_se(x)),
  data=fusion_data
)

kable(fix_agg_columns(stim_agg))
```

| movie | pct\_fusion\_mean | pct\_fusion\_se |
| --- | --- | --- |
| 1 | 14.3 | 1.6 |
| 2 | 48.5 | 2.2 |
| 3 | 26.1 | 2.2 |
| 4 | 39.8 | 2.6 |
| 5 | 21.5 | 2.1 |
| 6 | 52.6 | 2.3 |
| 7 | 62.4 | 2.4 |
| 8 | 64.1 | 2.3 |
| 9 | 83.4 | 1.7 |

```
by_group <- stim_agg <- aggregate(
  pct_fusion ~ language + movie,
  function(x) round(d=1, m_se(x)),
  data=fusion_data
)

mm <- matrix(nrow=2, byrow = F,
             by_group$pct_fusion[,1])


as_pdf('./barplot_by_stim_fusion', w=1.33*4, h=2.5, {
  par(mar=c(2,3,1,1))
  xp <- barplot(mm,space=c(.125,1),
                beside=TRUE, border=cc, col=lighten(cc, 2/3),
                ylim = c(0,100), axes=F
  )
  ruta_axis(2, 0:2*50)
  legend('topleft', legend=c('CH', 'US'), text.col = colors[c('ch', 'us')], cex=2, bty='n')
  ebars.y(xp, by_group$pct_fusion[,1], by_group$pct_fusion[,2], col=cc)
})
```

## 3.3 LME on group x stimulus

```
fusion_data$subject_id %<>% factor
fusion_data$movie %<>% factor
fusion_data$language %<>% factor


re <- lmer(
  pct_fusion ~ language * movie + (1|subject_id), data=fusion_data
)

car::Anova(re)
```

```
## Analysis of Deviance Table (Type II Wald chisquare tests)
## 
## Response: pct_fusion
##                    Chisq Df Pr(>Chisq)    
## language          1.8939  1     0.1688    
## movie          1597.3987  8     <2e-16 ***
## language:movie  124.3913  8     <2e-16 ***
## ---
## Signif. codes:  0 '***' 0.001 '**' 0.01 '*' 0.05 '.' 0.1 ' ' 1
```

```
emmeans::emmeans(re, pairwise ~ language, by='movie')$contrasts
```

```
## movie = 1:
##  contrast estimate   SE   df t.ratio p.value
##  CH - US     -2.78 4.28 1078  -0.651  0.5154
## 
## movie = 2:
##  contrast estimate   SE   df t.ratio p.value
##  CH - US      1.70 4.28 1078   0.398  0.6906
## 
## movie = 3:
##  contrast estimate   SE   df t.ratio p.value
##  CH - US      1.54 4.28 1078   0.360  0.7188
## 
## movie = 4:
##  contrast estimate   SE   df t.ratio p.value
##  CH - US    -13.23 4.28 1078  -3.092  0.0020
## 
## movie = 5:
##  contrast estimate   SE   df t.ratio p.value
##  CH - US      3.88 4.28 1078   0.907  0.3646
## 
## movie = 6:
##  contrast estimate   SE   df t.ratio p.value
##  CH - US     -6.76 4.28 1078  -1.580  0.1143
## 
## movie = 7:
##  contrast estimate   SE   df t.ratio p.value
##  CH - US     28.68 4.28 1078   6.701  <.0001
## 
## movie = 8:
##  contrast estimate   SE   df t.ratio p.value
##  CH - US     17.79 4.28 1078   4.158  <.0001
## 
## movie = 9:
##  contrast estimate   SE   df t.ratio p.value
##  CH - US      6.73 4.28 1078   1.572  0.1162
## 
## Degrees-of-freedom method: kenward-roger
```

```
# get the effect size for just the language term
r0 <- MuMIn::r.squaredGLMM(lmer(
  pct_fusion ~ language + (1|subject_id) + (1|movie), data=fusion_data
))
cat("effect size for language term only: ", r0[1], ', as %: ', round(d=2, 100* r0[1]), '\n')
```

```
## effect size for language term only:  0.00222911 , as %:  0.22
```

```
r1 <- MuMIn::r.squaredGLMM(lmer(
  pct_fusion ~ language + movie + (1|subject_id), data=fusion_data
))

r2 <- MuMIn::r.squaredGLMM(lmer(
  pct_fusion ~ language * movie + (1|subject_id), data=fusion_data
))

cat("effect size for interaction term only: ", r2[1] - r1[1], ', as %: ', round(d=2, 100* (r2[1] - r1[1])), '\n')
```

```
## effect size for interaction term only:  0.0183834 , as %:  1.84
```
